# Short-term exposure to PM_2.5_ and vanadium and changes in asthma gene DNA methylation and lung function decrements among urban children

**DOI:** 10.1186/s12931-017-0550-9

**Published:** 2017-04-19

**Authors:** Kyung Hwa Jung, David Torrone, Stephanie Lovinsky-Desir, Matthew Perzanowski, Joshua Bautista, Jacqueline R. Jezioro, Lori Hoepner, Jamie Ross, Frederica P. Perera, Steven N. Chillrud, Rachel L. Miller

**Affiliations:** 1 Division of Pulmonary, Allergy and Critical Care of Medicine, Department of Medicine, College of Physicians and Surgeons, Columbia University, PH8E-101, 630 W. 168 St., New York, NY 10032 USA; 2 Division of Pediatric Pulmonary, Department of Pediatrics, College of Physicians and Surgeons, Columbia University, 630 W. 168 St., New York, NY 10032 USA; 3 Department of Environmental Health Sciences, Mailman School of Public Health, Columbia University, 722 W. 168 St., New York, NY 10032 USA; 4 Lamont-Doherty Earth Observatory, Columbia University, 61 Rt, 9 W Palisades, New York, 10964 USA; 5 Division of Pediatric Allergy, Immunology and Rheumatology, Department of Pediatrics, College of Physicians and Surgeons, Columbia University, PH8E-101, 630 W. 168 St., New York, NY 10032 USA

**Keywords:** Vanadium, PM_2.5_, Short-term exposure, DNA methylation, Overweight, Asthmatic children

## Abstract

**Background:**

Both short and long-term exposure to traffic-related air pollutants have been associated with asthma and reduced lung function. We hypothesized that short-term indoor exposure to fine particulate matter <2.5 μm (PM_2.5_) and vanadium (V) would be associated with altered buccal cell DNA methylation of targeted asthma genes and decreased lung function among urban children in a nested subcohort of African American and Dominican children.

**Methods:**

Six day integrated levels of air pollutants were measured from children’s homes (age 9–14; *n* = 163), repeated 6 months later (*n* = 98). Buccal samples were collected repeatedly during visits. CpG promoter loci of asthma genes (i.e., interleukin 4 (IL4), interferon gamma (IFNγ), inducible nitric oxide synthase (NOS2A), arginase 2 (ARG2)) were pyrosequenced and lung function was assessed.

**Results:**

Exposure to V, but not PM_2.5_, was associated with lower DNA methylation of IL4 and IFNγ. In exploratory analyses, V levels were associated with lower methylation of the proinflammatory NOS2A-CpG^+5099^ among asthmatic overweight or obese children but not nonasthmatics. Short-term exposure to PM_2.5_, but not V, appeared associated with lower lung function (i.e., reduced *z*-scores for forced expiratory volume in one second (FEV_1_, FEV_1_/ forced vital capacity [FEV_1_/FVC] and forced expiratory flow at 25–75% of FVC [FEF_25–75_]).

**Conclusions:**

Exposure to V was associated with altered DNA methylation of allergic and proinflammatory asthma genes implicated in air pollution related asthma. However, short-term exposure to PM_2.5,_ but not V, appeared associated with decrements in lung function among urban children.

**Electronic supplementary material:**

The online version of this article (doi:10.1186/s12931-017-0550-9) contains supplementary material, which is available to authorized users.

## Background

Both short-term and long-term exposure to fine particulate matter <2.5 μm (PM_2.5_) have been associated with reduced lung function [1–5]. However, pediatric cohort studies on short-term effects of PM_2.5_ on lung function are relatively scarce. Further, it is far from evident what components of PM_2.5_ cause these adverse health effects, and their underlying mechanisms. These components may include toxic agents like trace metals.

In the current study, we focused on vanadium (V) as the trace metal PM component because previous studies have shown that ambient levels of V, emitted from the burning of residual oil fuel mainly from residential heating and shipping ports [6] and traffic emissions [7], exhibited marked spatial variability in New York City (NYC) [8]. Measures of V have been associated with increased 1) cellular stress responses (i.e., Nuclear Factor kappa B) in human bronchial epithelial cells [9], 2) risk of PM_2.5_-related respiratory and cardiovascular hospitalizations [10], 3) mortality among elderly individuals [11], and 4) wheeze [12] and decreased lung function (i.e., forced vital capacity (FVC)) among children [13]. In the latter case, the V findings persisted after adjusting for co-pollutants (e.g., PM_2.5_ or elemental carbon (EC)), suggesting that V itself may be an important independent contributor of adverse respiratory effects of PM_2.5_. Moreover, susceptibility to these pollutants may vary by asthma phenotype, including obesity-related asthma [14, 15]. Overweight asthmatics exhibited more asthma-like symptoms in association with exposure to PM_2.5,_ nitrogen dioxide (NO_2_) and polycyclic aromatic hydrocarbons (PAH) [16, 17], and greater declines in lung function in association with exposure to ozone [14].

Environmental epigenetic regulation also may underlie mechanisms of air pollution-associated asthma [18, 19]. As examples, recent ambient PM_2.5_ levels were associated with lower DNA methylation of the proinflammatory gene inducible nitric oxide synthase (iNOS encoded by NOS2A) [20], and chronic exposure to PAH was associated with methylation of the asthma regulatory gene Forkhead box transcription factor 3 (FOXP3) [21]. In a study of boilermakers, higher occupational levels of PM_2.5_, presumably representing high levels of metals [22], were associated positively with methylation of long interspersed nuclear element-1 (LINE-1) in peripheral blood leukocytes [23], indicating higher global methylation. However, pediatric cohorts have not yet investigated epigenetic changes of asthma genes in response to measures of air pollutants, and specifically for the V component.

Our objective was to delineate the association between residential exposure to air pollution, including PM_2.5_ and its metal component V, on epigenetic regulation and lung function in a nested cohort of asthmatics and healthy urban children. We specifically targeted epigenetic loci previously implicated in air pollution-related asthma [24–27]. We also quantified DNA methylation levels in buccal cells, aerodigestive track epithelium where air pollution-related molecular changes have been documented [28], via pyrosequencing technology to capture small differences [29–32]. We hypothesized that exposure to PM_2.5_ and V, assessed by residential measures integrated over 6 days, repeated 6 months later, would be associated with changes in buccal cell DNA methylation of targeted CpG loci in the promoter region of several asthma inflammatory genes (e.g., interleukin 4 (IL4), interferon gamma (IFNγ), NOS2A and arginase2 (ARG2)) among urban African American and Dominican children. Further, we explored whether such methylation would vary by obesity-asthma. We also hypothesized that short-term residential exposure to PM_2.5_ and V would be associated with decreased lung function.

## Methods

### Study population and residential air monitoring

Seven hundred twenty seven nonsmoking mothers of African-American and Dominican ethnicity living in Northern Manhattan and the South Bronx were recruited during pregnancy as part of the Columbia Center for Children’s Environmental Health cohort (CCCEH) birth cohort [33]. For this nested study, participants were recruited in order based on age criteria (9–14 years old still enrolled in CCCEH) and enriched for current asthma status (57% asthmatic vs 43% non-asthmatic). Children were classified as asthmatic if a specialized physician (allergist, pulmonologist) diagnosed them with asthma using study standardized and objective criteria, and if they had symptoms or used asthma medication in the 12 months prior to enrollment in the nested study [34]. Children without any asthma-related symptoms between age 5 and enrollment, or determined not to be asthmatic by our standardized criteria [34], in the nested study were classified as non-asthmatic. Height, weight, and body fat percentages (%BF) were measured at each visit (Time 1 and Time 2, 6 months later) using a portable stadiometer (SECA, Hamburg, Germany) and a segmental body composition monitor (Tanita Corporation, Tokyo, Japan). Children with body mass index (BMI) ≥ the age- and sex-specific 85^th^ percentile of the year 2000 CDC growth charts for age and sex were classified as ‘overweight’ [35]. Waist circumference (WC) was measured twice at a level midway between the top of the hip bone and the lowest rib. High %BF and high WC, classified as the upper 33% of mean whole %BF and mean WC, respectively, were further investigated based on the strong correlations between BMI and %BF or WC in studies with young adolescents [36, 37].

**Fig. 1:**
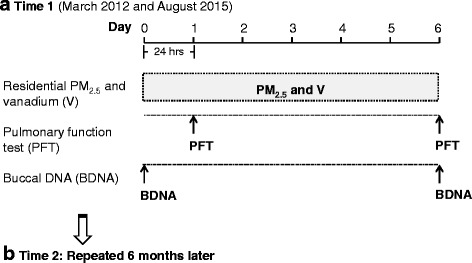
Study design. Residential indoor monitoring, pulmonary lung function test, and buccal sample collection over a 6 day sampling period, repeated 6 months later, are displayed. **a** Time 1 (March 2012 and August 2015). **b** Time 2: Repeated 6 months later

**Fig. 2:**
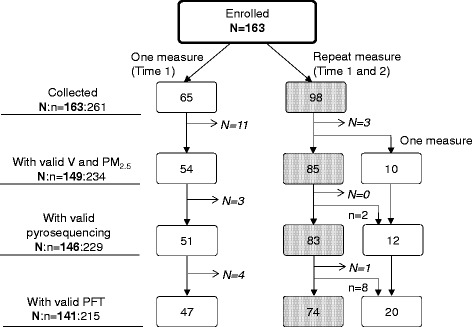
Schematic demonstration of study data. Numbers in the box represent the number of participants. N:*n* = number of repeat subjects: number of observations. Grey dotted box indicates two measures (both Time 1 and Time 2, 6 months apart) available and white box only one measure (Time 1) available. *N* = 14 participants dropped due to invalid residential air pollution data, resulting in *n* = 234 (54 + 85 × 2 + 10) data points available. *N* = 3 participants (equivalent to *n* = 5 observations as two participants with repeat measure had one invalid data) were excluded from the analysis of DNA methylation due to technical failures in the laboratory and *N* = 5 more participants (equivalent to *n* = 14 observations) were removed due to invalid pulmonary function tests

Indoor air monitors collected six-day integrated PM_2.5_ filter samples at each of the 163 homes between March 2012 and August 2015 (Time 1; initial set-up) (Fig. 1). Samplings started on Wednesday or Thursday to minimize variation in air pollution exposure by day of the week [38]. The sampling was repeated approximately six months after the initial sampling period (Time 2; *n* = 98) to capture the seasonal variability in air pollution levels. Residential indoor monitors were placed in a room where the child spent most of his or her time. Data were analyzed for those children (*n* = 149) for whom measures of residential PM_2.5_ and V were available (Fig. 2). In order to perform a sensitivity analysis including chronic exposure to PM_2.5_, we obtained residential indoor PM_2.5_ data that were collected 4–8 years prior to Time 1, using the same sampling methodology. Of 163 children, 106 children had previously available PM_2.5_. The study was approved by the Columbia University Institutional Review Board and written informed consent and assent were obtained.

### Residential indoor assessment

PM_2.5_ was analyzed by weighing (post-pre weight) Teflon filter samples collected from a cyclone with a 2.5 μm aerodynamic-diameter cut point (model SCC 1.062, BGI, Inc.) that operated at 1.5 L/min (±15% of standard deviation; SD) for six days. V levels on the same filters were analyzed using XEPOS Energy-Dispersive X-Ray Fluorescence Spectrometer (XEPOS 3, Spectro, Kleve, Germany) in specially-designed polytetrafluoroethylene (PTFE) holders to keep the filters in a fixed flat geometry [39]. The XEPOS had been calibrated for PM_2.5_ filters for V and other metals. Filter blanks and an internal standard consisting of a NYC PM_2.5_ filter were counted with each analysis batch of 10 filters.

### Buccal sample collection, DNA extraction and methylation

Buccal samples were collected during in-home visits on Day 0 (set-up day) and Day 6 (take-down day; Fig. 1) to capture changes in DNA methylation over 6 days. The participants were asked to rinse out their mouths with water before buccal swabs sampling to avoid contamination from food and then brush the inside of each cheek for fifteen seconds with a CytoSoft cytology brush (Fischer Scientific, Pittsburgh, Pennsylvania). The brushes were placed immediately in cell lysis solution (Qiagen Sciences, Germantown, Maryland) and stored at room temperature until processing. To ensure a homogenous cell population, brush smears were generated from twenty-five participants selected at random and stained using hematoxylin and eosin. Eight fields were randomly selected and imaged at 10X using a Nikon Eclipse TS100 (Tokyo, Japan) brightfield microscope. Two individuals independently counted and determined that 94% percent of the total cells were squamous epithelial cells among the 24 participants (kappa agreement score 0.934).

DNA was extracted and isolated using the Gentra Puregene Buccal Cell kit (Qiagen), qualified and quantified using a NanoDrop spectrophotometer (Thermo Scientific, Waltham, MA). Extracted DNA was bisulfite converted with the EZ-96 DNA Methylation-Lightning Kit (Zymo Research, Irvine, CA). Targeted promoter region CpG loci were selected based on their known importance to allergy and to NOS2A-related inflammation [25, 40], and published epigenetic links to asthma-related environmental exposures and/or asthma outcomes (Additional file 1: Figure S1 and Table S1). PCR primers were designed for the targeted regions using Pyromark Assay Design SW 2.0 Software (Qiagen) (Additional file 1: Table S2). PCR was performed using Qiagen HotStarTaq DNA Polymerase (Qiagen), and methylation levels for each of the targeted CpGs was measured using the Pyromark Q96 MD pyrosequencing instrument (Qiagen).

Quality assurance measures included: 1) methylated and unmethylated DNA (Qiagen) was added to each PCR plate as a control, and 2) the methylation level of a duplicate buccal sample collected at the same time was compared for 13% of the cohort (*n* = 23-29). The average absolute percent differences for the IL4, IFNγ, and NOS2A measures between the primary and duplicate buccal sample ranged between 2% and 7%, indicating a good agreement. The ARG2 loci exhibited greater average percent differences, ranging from 54% to 76% for CpG ^-30^ and CpG^−32^, respectively. One additional data point was excluded due to PCR contamination.

### Pulmonary function tests (PFTs)

PFTs were conducted during in-home visits on Day 1 and Day 6 using a portable spirometer (Koko, nSpire Health, Longmont, Colorado), in accordance with ATS and ERS guidelines [34] and repeated 6 months later (Fig. 1). Tests were considered acceptable if they met the following criteria: 1) rapid upstroke, 2) volume extrapolated <5% of FVC, 3) minimal premature termination of exhalation (premature termination = termination at >15% of peak flow), and 4) smooth exhalatory limb [41], as determined by two pulmonologists. PFTs that did not meet the acceptability criteria were excluded (*n* = 14 out of 229; Fig 2). Four spirometry outcome measures were included for analysis: FVC, forced expiratory volume in one second (FEV_1_), FEV_1_/FVC, and forced expiratory flow at 25–75% of forced vital capacity (FEF_25–75_).

### Statistical analyses

Chi-square and Mann–Whitney tests were used to detect the difference in demographic characteristics between groups and air pollutant levels by heating season (i.e. October-April), respectively. Consideration of heating season was to assess meteorological conditions such as temperature, humidity, and cold/flu season which could confound measures of air pollution and respiratory morbidity [12, 42, 43]. A spearman correlation coefficient was computed for correlations between PM_2.5_, and V while the intraclass correlation coefficient (ICC) was calculated for correlations among repeated measures of percent methylations. Air pollutant concentrations were log-transformed to assume normal distribution for subsequent analyses.

Due to non-normal distribution of the log-transformed methylation data, percent methylation of IL4, IFNγ, and NOS2A were dichotomized at the upper tertile of each individual CpG site [44]. Percent methylation of ARG2 were averaged across 3 selected CpG sites then further dichotomized as ‘unmethylated (0)’ vs ‘methylated (1)’ if the average percent methylation was zero or > 0, as described [25]. The associations among residential levels of PM_2.5_, V, and changes in DNA methylation were analyzed using a modified Poisson regression in generalized estimating equations (GEE) models to estimate relative risks (RR) [45]. The analyses were conducted using PM_2.5_ and V (two-pollutant models), and DNA methylation on Day 6 with adjustment of Day 0 DNA methylation to assess the independent effects of each pollutant on changes in DNA methylation. Final models were further adjusted for common covariates, including race/ethnicity, sex, age, heating season, asthma diagnosis, and overweight. Exploratory analyses were conducted to examine differences in air pollution-related DNA methylation by overweight asthmatic phenotype, suggested in previous in cross-sectional studies [26, 46]. Adjusted models stratified by overweight and asthma (i.e., 4 groups: overweight asthmatics, non-overweight asthmatics, overweight non-asthmatics, and non-overweight non-asthmatics) were run. A similar analysis was performed after replacing overweight with obesity (BMI ≥ the age- and sex-specific 95^th^ percentile), high BF%, or high WC.

Spirometric variables were converted to ethnic-specific *z*-scores, which were adjusted for sex, age, and height, according to the Global Lung Initiative (GLI-2012) equations for African American and Dominican (mixed ethic origin treated as ‘other’) children using the GLI-2012 software for subsequent analyses (http://www.ers-education.org/guidelines/global-lung-function-initiative/tools.aspx) [47]. Multivariable linear regression analyses in two-pollutant models were used to examine the observed effects of PM_2.5_ on each lung function outcome on Day 6, after controlling for V, Day 1 lung function outcome, heating season, asthma diagnosis, and overweight. Of the 149 children with valid air monitoring data, 3 and 8 children, respectively, were excluded due to invalid methylation (i.e. failed pyrosequencing run) and lung function data, resulting in a final sample of 146, and of 141 for the DNA methylation and lung function analysis, respectively (Fig. 2).

Sensitivity analyses were conducted as follow: (1) reanalysis after controlling for time-spent at home to address the impact of residential exposure vs other microenvironments (e.g., school and outdoors), (2) reanalysis after replacing concurrent PM_2.5_ levels with those measured 4–8 years prior to Time 1, as a surrogate for chronic exposure to PM_2.5_ in methylation analysis, (3) reanalysis after controlling for food intake, by asking the question “In the past two hours, have you had anything to eat or drink? (Yes/No)”, and (4) reanalysis after replacing heating season with four seasons (i.e., spring, summer, fall and winter), given the reported seasonal variation in lung function outcomes [48]. All analyses were performed using SPSS Statistic version 23.0 (SPSS Inc., Chicago, IL, USA) where p < 0.05 was considered statistically significant.

## Results

### Subject characteristics and residential exposure

**Fig. 3:**
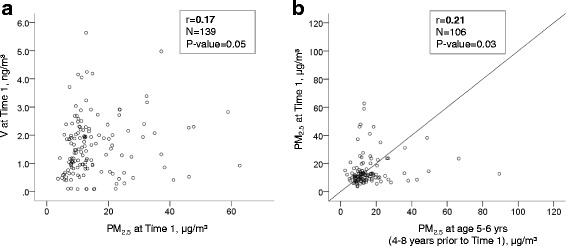
Correlations between (**a**) PM_2.5_ and V measured at Time 1, and (**b**) repeated residential indoor measures of PM_2.5,_ 4–8 years apart Of 139 children who had valid air pollution data at Time 1, 106 had previous residential indoor measurements that were collected 4–8 years ago using the same sampling methodology

There were no significant differences in demographic variables by enrollment into the nested cohort, except for a higher proportion of asthma and seroatopy, consistent with our recruitment strategy (Table 1). On daily average, children spent 68% (16.3 h) of their time at home. At Time 1, children were exposed to the median levels (Interquartile range IQR) of 11.9 (9.1) μg/m^3^ and 1.44 (1.34) ng/m^3^ of residential PM_2.5_ and V, respectively. A significant seasonal pattern was detected for V. Levels of V, but not PM_2.5,_ were higher during the heating compared to the nonheating season (Additional file 1: Figure S2). While residential levels of PM_2.5_ at Time 1 moderately correlated with those at Time 2, 6 months later, V weakly correlated with repeated measures, possibly due to substantial seasonal variations (Additional file 1: Figure S3). PM_2.5_ levels weakly correlated with V levels (Fig. 3). Furthermore, indoor levels of PM_2.5_ at Time 1 weakly correlated with those measured 4–8 years prior to Time 1, on the same children (Fig. 3), suggesting common chronic sources of PM_2.5_ air pollution.

**Table 1:** Cohort characteristics

Characteristic	Participants included^a^ (*n* = 149)	CCCEH cohort not included (*n* = 578)	*P*-value^h^
Maternal ethnicity			0.57
Dominican	94/149 (63%)	379/578 (66%)	
African American	55/149 (37%)	199/578 (34%)	
Age mean [min-max], yrs	12.5 (9.2–14.3)	-	
Girls	76/149 (51%)	300/578 (52%)	0.85
≥Maternal high school degree	82/144 (57%)	374/569 (66%)	0.05
Maternal asthma (+)	42/149 (28%)	121/578 (21%)	0.06
Prenatal ETS exposure^b^ (+)	48/147 (33%)	198/570 (35%)	0.64
Current ETS exposure^c^ (+)	12/121 (10%)	-	
Daily time spent home^d^ hrs, mean ± SD	16.3 ± 4.8		
BMI^e^ z score, mean ± SD	0.86 ± 1.11	-	
Underweight (<5^th^ %ile)	5/149 (3%)	-	
Normal weight (5^th^–85^th^ %ile)	66/149 (44%)	-	
Overweight (85^th^–95^th^ %ile)	40/149 (27%)	-	
Obesity (≥95^th^ %ile)	38/149 (26%)	-	
% Body fat, mean ± SD	26.7 ± 8.4	-	
Waist circumference cm, mean ± SD	71.5 ± 11.5	-	
Asthma^f^	87/149 (58%)	83/360 (23%)	<0.001
Seroatopy^g^	74/134 (55%)	113/269 (42%)	0.01

*CCCEH* Columbia Center for Children’s Environmental Health, *BMI* Body mass index, ETS: Environmental tobacco smoke, IgE: Immunoglobulin E, SD: standard deviation

The total number of CCCEH cohort participants enrolled at birth = 727; ^a^Includes only the children in nested study that had complete data available for current analysis. Participants excluded if residential indoor sampling data not collected due to entry criteria (*n* = 564) and invalid air pollution data (*n* = 14)

^b^Report of any smoker in the house during pregnancy. All mothers were nonsmokers during pregnancy

^c^Report of any smoker during 1-week sampling period

^d^Determined by 24-hour questionnaire by answering “Between the time we dropped off the monitor and when you went to bed, did you leave home (Yes/No); then “What time did you leave home?”; “What time did you get home?”

^e^Weight (kg)/height (m)^2^, SD, standard deviation

^f^Determined by a specialist physician using standardized criteria at age 5–12 year [34]

^g^Total IgE ≥ 80 IU/mL

^h^Chi-tests performed

### Buccal cell asthma gene methylation

In general, the targeted CpG sites in the IL4 promoter were heavily methylated, while the CpG sites in ARG2 were largely unmethylated; the latter is consistent with previous findings in buccal cells (Additional file 1: Figure S4) [25]. IFNγ methylation levels also were comparable to those reported in other urban cohorts [49]. Within each gene loci, two targeted CpG sites weakly correlated (Additional file 1: Figure S5). In general, repeated measures (Day 0 and Day 6) of percent methylation showed low values of ICCs (Additional file 1: Table S3), indicating substantial within-subject variability in methylation levels over the short-term, as previously described [50].

### Associations among PM_2.5_, V exposures and DNA methylation

Overall, PM_2.5_ levels were not associated with DNA methylation at the CpG loci in IL4, IFNγ, NOS2A, and ARG2 gene in two-pollutant models (Table 2). In comparison, the relative risk of higher (i.e. upper tertile) IL4 CpG^−326^ and IFNγ CpG^−54^ methylation decreased with higher residential V exposure in adjusted models (Table 2). Associations between V exposure and DNA methylation in NOS2A and ARG2 loci were not significant.

**Table 2:** Associations between residential measures of PM_2.5_, V and Day 6 DNA methylation in adjusted models (N:*n* = 146:229)

		RR_adj_^a^ [95% CI]	
Gene	CpG sites	PM_2.5_	V
IL4	−326	1.16 [0.84–1.61]	0.80 [0.65–0.98]^*^
	−48	1.04 [0.76–1.43]	1.05 [0.83–1.34]
IFNγ	−186	0.87 [0.64–1.19]	1.23 [0.96–1.58]
	−54	1.03 [0.79–1.35]	0.81 [0.67–0.98]^*^
NOS2A	+5099	1.07 [0.79–1.45]	0.97 [0.80–1.19]
	+5106	1.07 [0.74–1.54]	0.98 [0.78–1.23]
ARG2	−32, −30, and −26^b^	1.07 [0.97–1.17]	0.96 [0.89–1.02]

N: number of repeat subjects included for the analysis and n: number of observations from both Time 1 and Time 2

^a^Adjusted for race/ethnicity, sex, age, heating season, asthma diagnosis, overweight, and DNA methylation at Day 0 (Two-pollutant models)

^b^Average of ARG2 CpG sites of −32, −30, and −26

^*^ *p*-value < 0.05

In exploratory analyses that stratified by overweight and asthma (i.e., four groups), there were still no significant associations between PM_2.5_ and DNA methylations in the targeted asthma genes (Additional file 1: Table S4). In comparison, a distinct methylation pattern following V exposure was observed among the 4 groups. A significant association between V levels and lower methylation of NOS2A CpG^+5099^ was observed among overweight asthmatic children (Additional file 1: Table S5). Interestingly, the opposite pattern (higher methylation) was observed among the overweight non-asthmatic children at the IFNγ CpG^−186^ locus. Further, among non-overweight asthmatic children, V levels were associated with lower methylation of IFNγ CpG^−54^ (Additional file 1: Table S5). Methylation of ARG2 and IL4 was not associated with V levels among any of the phenotypes. Furthermore, when overweight was replaced by obesity, the observed associations of V with DNA methylation of NOS2A CpG^+5099^, IFNγ CpG^−54^, and IFNγ CpG^−186^ shown in Additional file 1: Table S5, remained strong (RR [95% CI]: 0.40 [0.26-0.63]; *p* = 0.03, 0.71 [0.54-0.92]; *p* = 0.001, 1.42 [1.02-1.97]; *p* = 0.039 for NOS2A CpG^+5099^, IFNγ CpG^−54^, and IFNγ CpG^−186^ respectively). Interestingly, a non-significant association, previously observed between V and lower methylation of IL4 CpG^−326^ among overweight asthmatic children, became significant when overweight was replaced by obesity (RR [95% CI]: 0.40 [0.24-0.68]; *p* = 0.001), high-BF (RR [95% CI]: 0.47 [0.27- 0.80]; *p* = 0.006), or high-WC in asthmatic children (RR [95% CI]: 0.21 [0.08- 0.53]; *p* = 0.001).

### PM_2.5_ and V exposures and lung function

In multivariable linear regression models, residential PM_2.5_ levels, measured over 6 days, appeared associated with decreased *z*-scores for FEV_1_, FEV_1_/FVC and FEF_25–75_, including following adjustment for V levels (Table 3). In comparison, significant associations between V and lung function outcomes were not observed. Therefore, analysis for mediation by methylation of V on lung function was deferred.

**Table 3:** Associations between residential levels of PM_2.5_ and V and Day 6 lung function *z*-scores

Lung function	Beta coefficient ^a^ (95% CI) (N:*n* = 141:215)	
*z*-score^b^	PM_2.5_	V
FVC	−0.07 (−0.19, 0.05)	0.07 (−0.02, 0.15)
FEV_1_	−0.15 (−0.29, −0.01)*	0.09 (−0.02, 0.19)
FEV_1_/FVC	−0.17 (−0.31, −0.03)*	0.05 (−0.09, 0.19)
FEF_25–75_	−0.18 (−0.32, −0.04)*	0.03 (−0.12, 0.18)

N: number of repeat subjects included for the analysis and n: number of observations from both Time 1 and Time 2

*FVC* Forced vital capacity, *FEV*_*1*_ Forced expiratory volume in one second, *FEF*_*25*–*75*_ Forced expiratory flow at 25–75% of Forced Vital Capacity

^a^Two-pollutant models adjusted for heating season, asthma diagnosis, overweight, each lung function *z*- score measured on Day 1

^b^Based on reference equation from the Global Lung Initiative 2012 [47]

*p < 0.05

### Sensitivity analyses

First, after controlling for time spent home, the main findings in Table 2 remained similar although the association between V and methylation of IL4 CpG^−326^ became borderline significant (RR [95% CI]: 0.81 [0.66-1.00]; *p* = 0.052 for IL4 CpG^−326^ and 0.81 [0.67-0.97]; *p* = 0.026 for IFNγ CpG^−54^). Second, with an adjustment of residential chronic exposure, assessed by previous PM_2.5_ levels measured 4–8 years prior to Time 1, significant association between V and lower methylation of IFNγ CpG^−54^ in Table 2 remained with a smaller RR (RR [95% CI]: 0.76 [0.63-0.92]; *p* = 0.005) while the association between V and methylation of IL4 CpG^−326^ lost statistical significance (*p* = 0.10), possibly due to a smaller sample (N_[subjects]_ = 115 and n_[obervations]_ = 172). Third, when we controlled for food intake, the main findings in Table 2, persisted (Data not shown). Lastly, when the two heating vs nonheating season was replaced with four seasons, the significant associations of PM_2.5_ and lung function outcomes were replicated (data not shown).

## Discussion

In this nested cohort of African American and Dominican children living in NYC, we found that that 6 day-integrated residential V, but not PM_2.5_, was associated with lower buccal cell promoter DNA methylation of asthma T helper (Th) gene (i.e. IL4, IFNγ) loci, even after controlling for methylation levels 6 days previously. We also found that residential PM_2.5_ levels, but not V, were associated with lower lung function (i.e., *z*-scores for FEV_1_, FEV_1_/FVC and FEF_25–75_). To our knowledge, this is the first study to report altered asthma gene DNA methylation related to residential V exposure, and lung function decrements associated with short-term residential exposure to PM_2.5_ among urban children.

The strengths of this study include the 1) direct measurement of each child’s short-term (6 day) home exposure to air pollution, that may reduce misclassification of personal exposure and allow us to discern effects of individual key air pollutants (e.g., V and PM_2.5_), 2) use of repeat prospective measures of environmental air pollutants, DNA methylation, and lung function, which allow us to detect changes in each within the same children and consider seasonal effects (6 months apart), 3) targeted focus on the methylation of specific loci that previously were implicated in air pollution-related asthma. We did so by using pyrosequencing technology to capture small differences in their DNA methylation in order to validate the importance of these small differences to pediatric urban asthma, and 4) utilization of a well-phenotyped prospective birth cohort study with detailed data on children’s clinical status and past residential PM_2.5_ levels; the latter of which correlated over a 4–8 year time period (Fig. 3) and allowed us to use as a surrogate for residential chronic exposure to PM_2.5_ in sensitivity analyses.

We chose to focus on altered methylation of mechanistically relevant gene loci that were implicated previously in air pollution-related asthma, given the emerging environmental epigenetic literature (Additional file 1: Table S1). In cohort studies of electric furnace steel plant workers, boilermaker welders, chronic obstructive pulmonary disease (COPD) patients in China, and children in Southern California, exposure to PM_2.5_ was associated with altered NOS2A promoter region DNA methylation [20, 27, 51, 52], suggesting environmental epigenetic mechanisms. We found that residential V, but not PM_2.5_, was associated with lower methylation of several Th gene promoter loci, including IL4 CpG^−326^ and IFNγ CpG^−54^. The findings may be supported by observations in mouse CD4+ splenic T cells following concomitant exposure of *Aspergillus fumigatus* and diesel exhaust particles (DEP) [24], mouse CD4+ T cells from lung-draining lymph nodes following chronic ovalbumin challenge [53], and numerous human cohort experimental studies [54] (Additional file 1: Table S1). Because DNA methylation in promoter regions is usually critical to gene silencing in human cells [55], its decrease is consistent with upregulated expression of the proallergic immune response. In comparison, lower methylation of IFNγ CpG^−54^, while considered important to suppressing allergic immune responses in some mouse models [56], also has been observed paradoxically to enhance allergy or airway hyperresponsiveness [53, 57]. We did not identify associations between residential PM_2.5_ and altered DNA methylation. One explanation may be the lack of short-term variability in PM_2.5_ compared to V, given the moderate correlation over time (Additional file 1: Figure S3), thus making it hard to capture a methylation signal due to smaller variations in PM_2.5_ levels. Alternatively, our results suggest that instead of PM_2.5_, a complex particle mixture of various chemical constituents from multiple sources, a specific individual metal component, V, may be one of the important drivers of epigenetic changes despite V only contributes an average of less than 1% to total PM_2.5_ mass.

Further, in exploratory analyses, we observed the associations between V and select DNA methylation differed by overweight asthma stratum. In particular, V was associated with lower methylation (and presumed greater gene activation) of NOS2A CpG^+5099^ only among overweight or obese asthmatic children. Also, the association between V and IL4 CpG^−326^ among overweight asthmatic children gained statistical significance when restricted to obese, high BF or high WC in asthmatics. These findings appear consistent with one small cross-sectional study that showed dysregulated DNA methylation in peripheral blood mononuclear cells (PBMCs) that varied by the asthma and obesity phenotype [46]. This group found that obese asthmatics, when compared to obese non-asthmatics, exhibited altered methylation in many pathways related to IL4, IFNγ and NOS2A gene functions (as shown in Additional file 1: Table S5), including IgE signaling, and interferon and chemokine activity [58, 59]. Interestingly, in the absence of asthma, the overweight or obese children were instead susceptible to hypermethylation of IFNγ CpG^−186^ following V exposure. This may be consistent with enhanced inflammation and differential regulation of T helper responses previously observed in obesity [60]. Despite the smaller sample size in stratified analysis, our results were consistent across overweight, obese, high BF and high WC classification, suggesting that obesity may enhance susceptibility to the effect of V on epigenetic changes.

Another objective was to evaluate adverse effects of a specific metal component of V on lung function in relation to PM_2.5_. Our findings that PM_2.5_ seemed to drive decrements in lung function are novel because 1) to date, no studies have looked at lung function in relation to short-term exposure in children. We did this by controlling for previous lung function *z*-scores measured on Day 1 to assess changes in lung function over a short period, and 2) we used direct measurement of PM_2.5_ through residential indoor monitoring, rather than estimates from land-use regression models employed in most studies [3, 61]. In comparison, counter to our prediction based on recent reports of V effects on lung function in children [13, 61], we were not able to detect any associations between residential V levels and lung function *z*-scores. But our study differed in terms of the shorter timeline of exposure to V and by the differences detected in the obstructive airway physiology characteristic of asthma (i.e., reduced FEV_1_), and not FVC.

The focus on quantifying differences in pre-selected CpG specific targets also allows us to compare their potential differential impact across loci, across a short timeline of exposure, and in limited case, across studies. For example, we observed relatively weak correlations between neighboring CpG sites within the same gene at Time 1 (Additional file 1: Figure S5). This observation may challenge previous reports that DNA methylation at adjacent CpG sites tends to display similar amounts of methylation [62]. However, they are consistent with emerging evidence in humans that suggest individual CpG sites may methylate to a different extent [49]. Another group also reported, in a controlled exposure study of allergen and diesel exhaust, substantial differences in single CpG site methylation in human bronchial epithelial cells [32]. This suggests that neighboring CpG sites may respond to exposures independently.

We acknowledge several limitations. First, methylation was measured in buccal cells, previously shown to inform on airway molecular changes [20, 25], and did not compare to the target lung tissue. However, lung tissue cannot be accessed repeatedly in children, and previous studies have documented high correlations in gene expression between tissues obtained from the buccal mucosa and lung [63, 64]. The cells themselves represent a relatively homogenous population of epithelial cells, as we demonstrated. Second, we monitored residential indoor air for exposure assessment, but not outdoor or school environments that may have important confounding effects. However, urban children in this study spent the majority (68% on daily average) of their time home, and studies have shown that outdoor PM_2.5_ and V readily penetrate indoors [65, 66]. Further, sensitivity analysis with an adjustment of time-spent home showed that our findings persisted. Third, we recognize that other CpG loci upstream or downstream of targets, and certainly other genes, may contribute to the air pollution epigenetic effects, and that the presence of single nucleotide polymorphisms (SNPs) could impact methylation levels. However, the effect sizes of variants measured in genome-wide association studies also seem small, driving our approach to capture additional small epigenetic responses. Last, other metal components of PM_2.5_ such as nickel and iron, that may important along with V in respiratory health [9, 12, 13], were not available. Nonetheless, with targeted epigenetic loci previously implicated in asthma and the use of pyrosequencing technology, we demonstrated for the first time that short-term residential V exposure is associated with changes in the degree of methylation of important asthma genes in children. This furthers our understanding of epigenetic regulation and its susceptibility to exposure to a specific air pollutant. Further, we explored associations between V and methylation by asthma-obesity and observed differential DNA methylation patterns by asthma phenotype. While intriguing, the interpretation of these results should be considered with caution due to a relatively small sample size in stratified analyses.

Additional discussions of the ambient air pollution levels, DNA methylation values, and the further study limitations are presented in Additional file 1.

## Conclusions

We found associations between short-term V exposure and DNA methylation of asthma gene loci. Short-term residential indoor measures of PM_2.5,_ but not V, may have been associated with lower lung function among urban children. While previous studies have suggested possible links between metal exposure and particulate matter and differences in DNA methylation pattern relevant to asthma, none to our knowledge have specified the key component of these pollutants. Further, to the best of our knowledge, this is the first study to investigate the effect of short-term V exposure on altered DNA methylation of asthma genes, and suggests that V may be important to urban asthma via epigenetic regulation. Although requiring further investigation in a large cohort, our results suggest asthma phenotypes may need to be considered in environmental and epigenetic studies of asthma. In turn these findings ultimately may help guide policy-related or medical interventions against specific pollutants, and against obesity, as well as provide methods for early identification of at-risk children.

## Additional file

Additional file 1: Table S1. Promoter region CpG locations and rationale. **Table S2.** Primers for PCR and pyrosequencing. **Table S3.** Intraclass correlation coefficients (ICC) among repeated measures of buccal cell DNA methylation. **Table S4.** Associations between residential PM_2.5_ and Day 6 DNA methylation: by asthma and overweight. **Table S5.** Associations between residential V and Day 6 DNA methylation: by asthma and overweight. **Figure S1.** Targeted CpG sites in promoter region. **Figure S2.** Seasonal variations in (a) PM_2.5_ and (b) vanadium (V). **Figure S3.** Repeated residential indoor measures of (a) PM_2.5_ and (b) vanadium (V)_,_ 6 months later. **Figure S4.** Distribution of percent DNA methylation of IL4, IFNγ, NOS2A, and ARG2 at Day 6. **Figure S5.** Correlation matrix for Day 6-buccal cell DNA methylations of IL4, IFNγ, NOS2A, and averaged ARG2 at Time 1. (DOCX 286 kb)

## Abbreviations

%BF: Body fat percentages
ARG: Arginase
BC: Black carbon
BMI: Body mass index
CCCEH: Columbia Center for Children’s Environmental Health
COPD: Chronic obstructive pulmonary disease
DEP: Diesel exhaust particle
EC: Elemental carbon
ETS: Environmental tobacco smoke
FEF_25–75_: Forced Expiratory Flow at 25–75% of Forced Vital Capacity
FeNO: Fractional exhaled nitric oxide
FEV_1_: Forced expiratory volume in one second
FOXP3: Forkhead box transcription factor 3
FVC: Forced vital capacity
GEE: Generalized estimating equation
GLI: Global lung initiative
ICC: Intraclass correlation coefficient
IFNγ: Interferon gamma
IL4: Interleukin 4
IgE: Immunoglobulin E
IQR: Interquartile range
LINE-1: Long interspersed nuclear element-1
NO_2_: Nitrogen dioxide
NOS2A: Inducible nitric oxide synthase
NYC: New York City
PAH: Polycyclic aromatic hydrocarbon
PBMCs: Peripheral blood mononuclear cells
PEFR: Peak expiratory flow rate
PFT: Pulmonary function test
PM: Particulate matter
PTFE: Polytetrafluoroethylene
RR: Relative risk
SNPs: Single nucleotide polymorphisms
V: Vanadium
WC: Waist circumference
