# Supplementary material for: Short-term exposure to PM2.5 and vanadium and changes in asthma gene DNA methylation and lung function decrements among urban children
Source: Respir Res. 2017 Apr 19;18:63. doi: 10.1186/s12931-017-0550-9 (PMC5397738; doi:10.1186/s12931-017-0550-9)
Supplement: Additional file 1: Table S1. — Promoter region CpG locations and rationale. Table S2. Primers for PCR and pyrosequencing. Table S3. Intraclass correlation coefficients (ICC) among repeated measures of buccal cell DNA methylation. Table S4. Associations between residential PM2.5 and Day 6 DNA methylation: by asthma and overweight. Table S5. Associations between residential V and Day 6 DNA methylation: by asthma and overweight. Figure S1. Targeted CpG sites in promoter region. Figure S2. Seasonal variations in (a) PM2.5 and (b) vanadium (V). Figure S3. Repeated residential indoor measures of (a) PM2.5 and (b) vanadium (V), 6 months later. Figure S4. Distribution of percent DNA methylation of IL4, IFNγ, NOS2A, and ARG2 at Day 6. Figure S5. Correlation matrix for Day 6-buccal cell DNA methylations of IL4, IFNγ, NOS2A, and averaged ARG2 at Time 1. (DOCX 286 kb) [file 12931_2017_550_MOESM1_ESM.docx]

**Additional file 1**

**Table S1.** Promoter region CpG locations and rationale

| Gene | CpG sites | Rationale |
| --- | --- | --- |
| IL4 | -326 | - Conserved in mice as CpG^-393^ [1, 2]  - Underwent altered CD4+ T cell methylation in mice following prenatal mold allergen and following ovalbumin [1, 2] |
|  | -48 | - Associated with the diagnosis of pediatric asthma in two independent inner-city cohorts[3] |
| IFNγ | -186/ -54 | - Conserved in mice (CpG^-186^ as CpG^-190^; CpG^-54^ as CpG^-190^) [1, 2]  - Methylation correlated negatively with gene expression [4-6], and sufficient to abrogate IFNγ gene activity [7]  - Altered methylation associated with exposure to allergen and diesel in mice [2, 5, 8]  - Altered methylation associated with exposure to traffic-related air pollution and secondhand smoke in children [6], including CCCEH cohort [9]  - Altered methylation associated with allergy and asthma [5, 8, 10] |
| NOS2A | +5099 | - Associated with PM-induced demethylation in buccal cells in children [11], and J_NO_, a correlate of fractional exhaled nitric oxide (FeNO) and seroatopy, in CCCEH cohort [12] |
| ARG2 | -32^§^/-30/-26^§^ | - CpG^-32^ conserved in mice as CpG^-54^  - Regulated gene expression [13, 14]  - Exhibited altered buccal cell methylation in association with airway inflammation among asthmatic children [15] |

**Table S2**. Primers for PCR and pyrosequencing

| Gene / CpG | Forward | Reverse | Sequencing |
| --- | --- | --- | --- |
| IL–4 / -326 | 5’– [Biotin] – TGTTTTGTATAGAAGGGAGAGGTTATAG – 3’ | 5’ – TTCCCCAAAAAAACTACATTACAAC – 3’ | 5’– CCCCAAAAAAACTACATT – 3’ |
| IL–4 / -48 | 5' – GTTGATTGGTTTTAAGTGATTGATAATT – 3' | 5 '– [Biotin] – ACTAACCATACAATACTAACAAAAATATCT – 3' | 5' – AATTTTTAATGTAAATTTATTTTTT – 3' |
| IFNγ / -186 | 5’– [Biotin] – AGATGGTGATAGATAGGTAGGGATGATA–3’ | 5’ – TCCCACCAAAATAACACAAATAAACAT – 3’ | 5’ – AAATAAACATAATAAATCTATCTCA –3’ |
| IFNγ / -54 | 5’ – ATGTGTTGTATTTTTTTTGGTTGTTGGTAT – 3’ | 5’– [Biotin] – TATCATCCCTACCTATCTATCACCATCTC – 3’ | 5’– ATTGAAGTTTTTTGAGGATT – 3’ |
| NOS2A / +5099; +5106 | 5’ – AGTGTTTTTTAGGGTTAGGTAAAGGTAT – 3' | 5’– [Biotin] – CCAACCAAACTTCATCACTAACCAC – 3’ | 5’ – GGGTTAGGTAAAGGTATT – 3’ |
| ARG2 / -32; -30; -26 | 5’ – GGGTTTGAGAAGAAGGTGTGT – 3' | 5’– [Biotin] – AAACTACCCCTTAAAAACATAATC – 3’ | 5’ – TGTAGGGGGTTGGTT – 3’ |

**Table S3.** Intraclass correlation coefficients (ICC) among repeated measures of buccal cell DNA methylation

| Gene | CpG promoter | ICCs on buccal samples Day 0 and Day 6 at Time 1^a^ |
| --- | --- | --- |
| IL4 | -326 | **0.20*** |
|  | -48 | -0.10 |
| IFNγ | -186 | **0.26**** |
|  | -54 | **0.18*** |
| NOS2A | +5099 | **0.26**** |
|  | +5106 | **0.35***** |
| ARG2 | -32 | -0.03 |
|  | -30 | 0.14 |
|  | -26 | 0.04 |
|  | -32, -30, -26^e^ | **0.18*** |

Note: N=134 (# of subjects)

^a^Time 1 is defined as the initial sampling set up

^e^Average methylation of ARG2 at CpG sites of -32, -30, and -26.

*p-value < 0.05, **p-value < 0.01 and ***p-value < 0.001.

**Table S4.** Associations between residential PM_2.5_ and Day 6 DNA methylation: by asthma and overweight

|  |  | RR_adj_^a^ [95% CI] | | | | |
| --- | --- | --- | --- | --- | --- | --- |
|  |  | Asthma | |  | No asthma | |
| Gene | CpG Sites | Overweight | Non-overweight |  | Overweight | Non-overweight |
| # subjects (N) | | 46 | 43 |  | 36 | 31 |
| Number of observations (n) | | 65 | 66 |  | 53 | 45 |
| IL4 | -326 | 1.49 [0.77-2.92] | 0.80 [0.54-1.19] |  | 1.27 [0.75-2.15] | 1.28 [0.54-3.03] |
|  | -48 | 1.55 [0.75-3.20] | 0.94 [0.51-1.71] |  | 1.01 [0.52-1.95] | 1.42 [0.53-3.85] |
| IFNγ | -186 | 1.30 [0.61-2.76] | 0.84 [0.54-1.32] |  | 0.96 [0.53-1.75] | 0.56 [0.16-1.97] |
|  | -54 | 1.08 [0.54-2.16] | 1.21 [0.82-1.79] |  | 0.95 [0.60-1.51] | 0.99 [0.36-2.71] |
| NOS2A | +5099 | 0.72 [0.38-1.38] | 1.21 [0.72-2.04] |  | 1.27 [0.59-2.75] | 0.75 [0.26-2.14] |
|  | +5106 | 1.06 [0.56-2.00] | 1.10 [0.50-2.42] |  | 0.72 [0.30-1.72] | 1.69 [0.31-9.12] |
| ARG2 | -32, -30, -26^b^ | 1.07 [0.89-1.29] | 1.06 [0.77-1.45] |  | 1.07 [0.95-1.21] | 1.20 [0.82-1.75] |

^a^Model adjusted for race/ethnicity, sex, age, heating season, Day 0 methylation, and V (Two-pollutant models).

^b^Average methylation of ARG2 at CpG sites of -32, -30, and -26.

**Table S5.** Associations between residential V and Day 6 DNA methylation: by asthma and overweight

|  |  | RR_adj_^a^ [95% CI] | | | | |
| --- | --- | --- | --- | --- | --- | --- |
|  |  | Asthma | |  | No asthma | |
| Gene | CpG Sites | Overweight^b^ | Non-overweight |  | Overweight^b^ | Non-overweight |
| # of subjects (N) | | 46 | 43 |  | 36 | 31 |
| Number of observations (n) | | 65 | 66 |  | 53 | 45 |
| IL4 | -326 | 0.74 [0.46-1.18] | 1.09 [0.73-1.65] |  | 0.78 [0.59-1.03] | 0.64 [0.23-1.77] |
|  | -48 | 0.77 [0.57-1.03] | 1.33 [0.77-2.32] |  | 1.32 [1.04-2.11] | 0.50 [0.23-1.12] |
| IFNγ | -186 | 0.86 [0.56-1.31] | 1.26 [0.81-1.95] |  | **1.79 [1.30-2.46]***** | 0.88 [0.33-2.34] |
|  | -54 | 0.85 [0.66-1.09] | **0.62 [0.44-0.89]*** |  | 0.72 [0.47-1.08] | 0.68 [0.23-1.95] |
| NOS2A | +5099 | **0.68 [0.51-0.91]*** | 1.08 [0.75-1.56] |  | 1.27 [0.75-2.13] | 1.08 [0.49-2.37] |
|  | +5106 | 1.02 [0.68-1.52] | 1.04 [0.64-1.70] |  | 0.97 [0.69-1.35] | 0.93 [0.21-4.18] |
| ARG2 | -32, -30, -26^c^ | 0.94 [0.84-1.04] | 1.00 [0.85-1.17] |  | 0.99 [0.89-1.10] | 0.73 [0.49-1.10] |

Note: N=10 children changed their overweight status between Time 1 and Time 2, resulting in increase in # of repeat subjects.

^a^Model adjusted for race/ethnicity, sex, age, heating season, BDNA on Day 0, and residential indoor PM_2.5_ (Two-pollutant models).

^b^BMI ≥ 85^th^ percentile; ^c^Average methylation of ARG2 at CpG sites of -32, -30, and -26; *p-value <0.05, and ***p<0.001.

**Figure S1.** Targeted CpG sites in promoter region

Promoter region CpG loci relative to the transcriptional start site (a) and translational start site (b).

Clear circle represents CpG site conserved in mice, black circle not conserved in mice, and grey circle not analyzed. CpG loci for ARG2 are located a CpG island while IL4, IFNγ, and NOS2A are located outside of a CpG island. CpG locations by gene:

**IL4** promoter sites analyzed: CpG^-326^ (conserved in mice as CpG^-393^ [1]), CpG^-48^; promoter sites not analyzed: CpG^-79^

**IFNγ** promoter sites analyzed: CpG^-186^ (conserved in mice as CpG^-190^ [2]), CpG^-54^ (conserved in mice as CpG^-53^ [1]); promoter sites not analyzed: CpG^-299^

**NOS2A** promoter sites analyzed: CpG^+5099^, CpG^+5106^ (NOS2A CpG^-359^ and NOS2A CpG^-352^ when numbered upstream from the initiation of mRNA coding of exon 1 [16]); promoter sites not analyzed: CpG^+5174^, CpG^+5219^, CpG^+5291^, CpG^+5313^, CpG^+5362^, CpG^+5371^

**ARG2** promoter sites analyzed: CpG^-32^ (conserved in mice as CpG^-54^ when counting upstream from the transcriptional start site), CpG^-30^, and CpG^-26^ [NCBI assembly for both: GRCm38.p4; location: NC_000078.6 (79130788..79156301)]); promoter sites not analyzed: CpG^-337^, CpG^-319^, CpG^-301^, CpG^-297^, CpG^-282^, CpG^-273^, CpG^-268^, CpG^-241^, CpG^-237^, CpG^-230^, CpG^-228^, CpG^-204^, CpG^-202^, CpG^-183^, CpG^-165^, CpG^-159^, CpG^-138^, CpG^-111^, CpG^-105^, CpG^-97^, CpG^-79^, CpG^-72^, CpG^-63^, CpG^-49^, CpG^-23^, CpG^-9^, CpG^-3^.

**Figure S2.** Seasonal variations in (a) PM_2.5_ and (b) vanadium (V)

Mann-Whitney test were performed to concentrations of residential PM_2.5_ and V measured during the heating vs nonheating season. The white and black lines show individual observations, while the white and black area show the distribution. The dotted line indicates the overall geometric mean and the thicker solid line shows the geometric mean concentration for each season. **p<0.01

**Figure S3.** Repeated residential indoor measures of (a) PM_2.5_ and (b) vanadium (V)_,_ 6 months later

N=85/149 of children had valid air pollution data at both Time 1 and Time 2. Spearman correlation coefficients are shown.

**Figure S4.** Distribution of percent DNA methylation of IL4, IFNγ, NOS2A, and ARG2 at Day 6

Boxplots of percent DNA methylation measured on Day 6 are presented. The line inside the box = medians; the box length = interquartile range (IQR); whiskers = the highest and lowest values that are not outliers (o, 1.5× IQR–3× IQR) or extreme values (*, >3× IQR).

**Figure S5.** Correlation matrix for Day 6-buccal cell DNA methylations of IL4, IFNγ, NOS2A, and averaged ARG2 at Time 1

The matrix was generated by using R software and size and color correspond to the correlation coefficient on Day 6.

**Online discussion**

**Air pollution levels:** While the study was not designed to compare V levels across cohorts, we observed that the residential V levels are 46-64% lower than those reported based on land-use regression models in Netherlands [17], 24-hr averaged residential and outdoor levels of V in Belgium [18], and long-term average concentrations of V in 106 US counties [19]. Residential indoor levels of V also were lower than earlier (2000-2007) one-week average levels measured from two outdoor monitoring sites in NYC [20]. One explanation is that increase use of cleaner fuels and phase-out of dirty fuel oils for heating by 2012 in NYC (by the Clean Heat Rule initiated in 2011) may result in reduction in ambient V levels, as shown in other air pollutants (i.e., black carbon (BC)/soot and PM_2.5_) monitored within the same cohort [21] as well as in New York City Community Air Survey [22]. We also observed significant higher levels of V in the heating season (35% higher than the nonheating season) when the use of residential heating oil is greater in NYC.

**DNA methylation:** We observed substantial changes in DNA methylation in 6 days, measured by low ICC values (**Table S3**), which is consistent with what we observed in the CCCEH cohort at younger ages over 4-7 days [16], and previous findings by others over 48 hours [23]. In addition, boilermaker welders experienced increases in NOS2A promoter region blood methylation between pre-and post-work one day shifts [24]. Given the limited published pediatric cohort data, it is more difficult to compare levels of measured CpG site-specific buccal DNA methylation with observations from other cohorts, although similar ranges for the IFNγ and ARG2 loci are documented in pediatric cohorts [15, 25]; the very low levels belonging to the latter is a common pattern in CpG islands [26]. The high levels of methylation observed at the IL4 promoter are consistent with patterns of higher methylation outside CpG islands [26]. Further, the low level of ARG2 methylation (e.g., ≥ 90% of data ranged between 0% and 3%) appears to mathematically drive the greater absolute percent difference between duplicate buccal samples.

**Additional study limitations:** 1) We did not correlate methylation levels with gene expression of our selected inflammatory genes. Nonetheless, measures of changes in methylation, even without known corresponding changes in expression, have been associated with relevant clinical outcomes in robust epidemiological studies [27, 28] and controlled experiments [2, 23]. 2) Due to the large expense of personal air pollution assessment and phenotyping over time employed, we did not retest in another cohort. Instead our approach was to validate findings on specific CpG loci previously found in other air pollution and asthma cohorts, diminishing the relevance of this concern. 3) We analyzed DNA methylation data as a dichotomous variable (at the upper tertile). Currently there is no standard and widely used approach to methylation in the literature. Although dichotomized analysis could miss some information from extracted from other methods, it has been used in recent methylation literature [15, 29]. 4) Despite the link between V exposure and DNA methylation, we did not observe its associations with lung function. Our primary objective was to determine the effect of short-term residential exposure to V on methylation levels of genes implicated in allergic asthma. These findings with respect to methylation point suggest that residential V may be regulating gene expression, even if there was not a statistically significant effect measured on lung function.

**References**

1. Collison A, Siegle JS, Hansbro NG, Kwok C-T, Herbert C, Mattes J, Hitchins M, Foster PS, Kumar RK: **Epigenetic changes associated with disease progression in a mouse model of childhood allergic asthma.** *Disease Models and Mechanisms* 2013, **6:**993-1000.

2. Niedzwiecki M, Zhu H, Corson L, Grunig G, Factor P, Chu S, Jiang H, Miller R: **Prenatal exposure to allergen, DNA methylation, and allergy in grandoffspring mice.** *Allergy* 2012, **67:**904-910.

3. Yang IV, Pedersen BS, Liu A, O'connor GT, Teach SJ, Kattan M, Misiak RT, Gruchalla R, Steinbach SF, Szefler SJ: **DNA methylation and childhood asthma in the inner city.** *Journal of Allergy and Clinical Immunology* 2015, **136:**69-80.

4. Gonsky R, Deem RL, Targan SR: **Distinct Methylation of IFNG in the Gut.** *Journal of interferon & cytokine research* 2009, **29:**407-414.

5. Brand S, Kesper DA, Teich R, Kilic-Niebergall E, Pinkenburg O, Bothur E, Lohoff M, Garn H, Pfefferle PI, Renz H: **DNA methylation of T H 1/T H 2 cytokine genes affects sensitization and progress of experimental asthma.** *Journal of Allergy and Clinical Immunology* 2012, **129:**1602-1610. e1606.

6. Kohli A, Garcia MA, Miller RL, Maher C, Humblet O, Hammond SK, Nadeau K: **Secondhand smoke in combination with ambient air pollution exposure is associated with increasedx CpG methylation and decreased expression of IFN-γ in T effector cells and Foxp3 in T regulatory cells in children.** *Clinical epigenetics* 2012, **4:**1.

7. Williams CL, Schilling MM, Cho SH, Lee K, Wei M, Boothby M: **STAT4 and T-bet are required for the plasticity of IFN-γ expression across Th2 ontogeny and influence changes in Ifng promoter DNA methylation.** *The Journal of Immunology* 2013, **191:**678-687.

8. Liu J, Ballaney M, Al-Alem U, Quan C, Jin X, Perera F, Chen L-C, Miller RL: **Combined inhaled diesel exhaust particles and allergen exposure alter methylation of T helper genes and IgE production in vivo.** *Toxicological Sciences* 2008, **102:**76-81.

9. Tang W-y, Levin L, Talaska G, Cheung YY, Herbstman J, Tang D, Miller RL, Perera F, Ho S-M: **Maternal Exposure to Polycyclic Aromatic Hydrocarbons and 5'-CpG Methylation of Interferon-[gamma] in Cord White Blood Cells.** *Environmental health perspectives* 2012, **120:**1195.

10. Runyon RS, Cachola LM, Rajeshuni N, Hunter T, Garcia M, Ahn R, Lurmann F, Krasnow R, Jack LM, Miller RL: **Asthma discordance in twins is linked to epigenetic modifications of T cells.** *PloS one* 2012, **7:**e48796.

11. Salam MT, Byun H-M, Lurmann F, Breton CV, Wang X, Eckel SP, Gilliland FD: **Genetic and epigenetic variations in inducible nitric oxide synthase promoter, particulate pollution, and exhaled nitric oxide levels in children.** *Journal of Allergy and Clinical Immunology* 2012, **129:**232-239. e237.

12. Kuriakose J, Rosa MJ, Perzanowski M, Miller R: **Bronchial nitric oxide flux may be better associated with inducible nitric oxide synthase promoter methylation.** *American journal of respiratory and critical care medicine* 2012, **185:**460-461.

13. Maarsingh H, Zaagsma J, Meurs H: **Arginase: a key enzyme in the pathophysiology of allergic asthma opening novel therapeutic perspectives.** *British journal of pharmacology* 2009, **158:**652-664.

14. Holguin F, Comhair SAA, Hazen SL, Powers RW, Khatri SS, Bleecker ER, Busse WW, Calhoun WJ, Castro M, Fitzpatrick AM, et al: **An Association between l-Arginine/Asymmetric Dimethyl Arginine Balance, Obesity, and the Age of Asthma Onset Phenotype.** *American Journal of Respiratory and Critical Care Medicine* 2013, **187:**153-159.

15. Breton CV, Byun H-M, Wang X, Salam MT, Siegmund K, Gilliland FD: **DNA methylation in the arginase–nitric oxide synthase pathway is associated with exhaled nitric oxide in children with asthma.** *American journal of respiratory and critical care medicine* 2011, **184:**191-197.

16. Torrone DZ, Kuriakose JS, Moors K, Jiang H, Niedzwiecki M, Perera F, Miller RL: **Reproducibility and intraindividual variation over days in buccal cell DNA methylation of two asthma genes, interferon γ (IFNγ) and inducible nitric oxide synthase (iNOS).** *Clinical epigenetics* 2012, **4:**1.

17. Bilenko N, Brunekreef B, Beelen R, Eeftens M, de Hoogh K, Hoek G, Koppelman GH, Wang M, van Rossem L, Gehring U: **Associations between particulate matter composition and childhood blood pressure—the PIAMA study.** *Environment international* 2015, **84:**1-6.

18. Jacobs L, Buczynska A, Walgraeve C, Delcloo A, Potgieter-Vermaak S, Van Grieken R, Demeestere K, Dewulf J, Van Langenhove H, De Backer H: **Acute changes in pulse pressure in relation to constituents of particulate air pollution in elderly persons.** *Environmental research* 2012, **117:**60-67.

19. Bell ML, Ebisu K, Peng RD, Samet JM, Dominici F: **Hospital Admissions and Chemical Composition of Fine Particle Air Pollution.** *American journal of respiratory and critical care medicine* 2009, **179:**1115.

20. Patel M, Hoepner L, Garfinkel R, Chillrud S, Reyes A, Quinn J, Perera F, Miller R: **Ambient Metals, Elemental Carbon, and Wheeze and Cough in New York City Children through Age 24 Months.** *American Journal of Respiratory and Critical Care Medicine* 2009, **180:**1107-1113.

21. Jung KH, Liu B, Lovinsky-Desir S, Yan B, Camann D, Sjodin A, Li Z, Perera F, Kinney P, Chillrud S: **Time trends of polycyclic aromatic hydrocarbon exposure in New York city from 2001 to 2012: Assessed by repeat air and urine samples.** *Environmental Research* 2014, **131:**95-103.

22. <http://www1.nyc.gov/assets/doh/downloads/pdf/environmental/comm-air-survey-08-14.pdf:> **The New York City Community Air Survey: Neighborhood air quality 2008 - 2014.** Accessed 08/31/2016.

23. Clifford RL, Jones MJ, MacIsaac JL, McEwen LM, Goodman SJ, Mostafavi S, Kobor MS, Carlsten C: **Inhalation of diesel exhaust and allergen alters human bronchial epithelium DNA methylation.** *Journal of Allergy and Clinical Immunology* 2017, **139:**112-121.

24. Kile ML, Fang S, Baccarelli AA, Tarantini L, Cavallari J, Christiani DC: **A panel study of occupational exposure to fine particulate matter and changes in DNA methylation over a single workday and years worked in boilermaker welders.** *Environmental Health* 2013, **12:**1.

25. Lovinsky-Desir S, Ridder R, Torrone D, Maher C, Narula S, Scheuerman M, Merle D, Kattan M, DiMango E, Miller RL: **DNA methylation of the allergy regulatory gene interferon gamma varies by age, sex, and tissue type in asthmatics.** *Clinical epigenetics* 2014, **6:**1.

26. Sormani G, Haerter JO, Lövkvist C, Sneppen K: **Stabilization of epigenetic states of CpG islands by local cooperation.** *Molecular BioSystems* 2016.

27. Michel S, Busato F, Genuneit J, Pekkanen J, Dalphin JC, Riedler J, Mazaleyrat N, Weber J, Karvonen A, Hirvonen MR: **Farm exposure and time trends in early childhood may influence DNA methylation in genes related to asthma and allergy.** *Allergy* 2013, **68:**355-364.

28. Murphy TM, Wong CC, Arseneault L, Burrage J, Macdonald R, Hannon E, Fisher HL, Ambler A, Moffitt TE, Caspi A: **Methylomic markers of persistent childhood asthma: a longitudinal study of asthma-discordant monozygotic twins.** *Clinical epigenetics* 2015, **7:**130.

29. Vidal AC, Semenova V, Darrah T, Vengosh A, Huang Z, King K, Nye MD, Fry R, Skaar D, Maguire R: **Maternal cadmium, iron and zinc levels, DNA methylation and birth weight.** *BMC Pharmacology and Toxicology* 2015, **16:**1.
